# Ophthalmology

**Published:** 1910-03

**Authors:** A. Ogilvy


### OPHTHALMOLOGY.

Of dealing with **chronic glaucoma,** like the making of books, there appears to be "no end." It was said long ago that the number of operations designed for the cure of entropium was some criterion of their success; so it is with chronic glaucoma. It seems almost impossible to prevent the wound healing closely with a subsequent return of the high tension. One reason doubtless for the non-success is that patients will not submit to operations until the disease is far advanced. At first the attacks are so insidious that little pain is produced; there is merely a narrowing of the field on the nasal side, with now and then a slight exacerbation, which is shown by a fog or halo round lights, or a smoky look in the air of a room. The central vision remains normal, often for years, and even when the field is cut down to a circle of about 10 or 20 degrees the central vision may still be 5/9 and with suitable glasses J1; still, the disease is there, and producing its untoward results. Of course, one must not forget that the cause of chronic glaucoma has not yet been determined. A wide difference of opinion exists between the Priestley Smith theory—that the root of the iris is crushed up against the cornea by the swelling lens in small hypermetropic eyes, thereby closing the meshes of the ligamentum pectinatum, through which the aqueous humour escapes into Schlemm's canal—and the Henderson theory—that the high tension is due to a sclerotising of the fibres, and consequent blocking of the ligamentum—and this may account for the partial success of the numerous operations; at any rate, it is admitted by nearly everyone that iridectomy, although it may do much good in acute glaucoma, is not by any means an entirely successful operation for the chronic form. Many ophthalmic surgeons have lately advocated newer methods; of these we may mention Legrange, Abadie, Herbert, Freeland Fergus, Elliott. There is a good deal of controversy at present as to who was first with these operations, and one hesitates to be drawn into the discussion. After all, what most surgeons want to know is, what the operation is and if it was successful, and not who did it or described it first. The main thing in operating is to get a filtrating scar which will allow escape of aqueous when the tension gets high. At first leaving a portion of iris in the wound and so preventing the close healing was advocated. This was found to lead to a subsequent cyclitic irritation, and owing sometimes to absorption of germs by the iris, caused a sympathetic ophthalmia, so that method was given up. Next came an operation for enclosing or tucking into the wound of a piece of conjunctiva turned in from above. Much difficulty was found in keeping the conjunctiva *in situ*. The conjunctiva was at one time stretched on to the back of the corneal flap, but naturally this did not turn out well. Next an incision was made after the manner of an ordinary cataract wound, but shorter, and with a long conjunctival flap. The top of the sclero-corneal flap was cut off with scissors, leaving a crescent-shaped aperture, which was covered with the replaced conjunctival flap. Following this came an operation done in the following manner[1]: the sclero-corneal wound as before with the conjunctival flap; then instead of cutting off the corneal part a piece of sclera was

[1] *Ophthalmoscope*, November, 1909.

punched out with a cutting forceps such as the rhinologists use, but very small. Finally, Fergus,[1] having lifted a conjunctival flap, used a trephine to perforate the sclera about 3 or 4 m.m. from the limbus. Following this, he pushed an iris repositor between the ciliary body and the sclera until he could see the point showing in the anterior chamber. He lays stress on this cyclodialysis, and says it is most important. Elliott[2] does not do cyclodialysis; he strips the conjunctiva down as far as he can, and makes his trephine hole straight into the anterior chamber. If it is necessary, that is to say if the iris bulges into his trephine hole, he excises the portion of iris protruded, so doing a tiny iridectomy. Both the trephining surgeons are greatly pleased with their operations, which they say are always successful, and have never failed to cause a lowering of tension which is permanent. They also lay stress on the ease of the operation compared with the others; in fact, Elliott says his operation is within the reach "even of a tyro." It will be a great relief to ophthalmic surgeons and their patients when some definite decision has been come to as to which is the best operation for this bugbear of a disease; still, more has been attained during the last two or three years than during the preceding fifty years.

**Cataract extraction,** or expression, as some would like to call it, is, unlike operations for chronic glaucoma, a singularly successful procedure; hence there are very few alterations to chronicle; in fact, there are only two worth mentioning. The first is concerned with the conjunctival flap. It has been found that where a conjunctival flap is left adhering to the corneal edge of the wound healing takes place sooner and is more effectual. The wound is also protected from the entrance of germs that may be present in the conjunctival sac. The disadvantage of this flap during the operation is that there is much bleeding, which unless constantly mopped up, thereby delaying the finish of the operation, obscures the view; furthermore, if the flap be turned down over the cornea, so as to be out of the way while the iridectomy is made, it prevents one seeing the points of the iris forceps and also the beak of the cystotome, which ruptures the lens capsule, so that one has to work in the dark and trust a good deal to Providence. Several operators—again the question of precedence crops up—suggest now that the conjunctival flap should not be completely cut through,[3] but that the upper end (the cornea having been divided) should be left adhering to the remainder of the conjunctiva and the lens expressed under this bridge, and so eventually extracted. Seeing that the ordinary results are so good, this seems to one an unnecessary complication to the operation; and although

[1] *Ibid.*, February, 1910. [2] *Ibid.*, December, 1909.
[3] *Ibid.*, November, 1909.

the suggestors, or rather, we might say, the resuscitators (for the operation was suggested by Czermak many years ago, but allowed to fall into disuse), claim brilliant results, the usual method is good enough for most people. The second improvement in cataract operation[1] is the revival of the McKeown method of washing out the lens debris from the anterior chamber. This is found to be a useful procedure in cases where the cataract is not quite ripe, and some clear debris may be left behind. The modification of McKeown's method is that, instead of using a syringe to either suck out the debris or wash it out, a constant stream is used from a douche, the raising or lowering of the receptacle for the warm saline solution, which is employed now, providing the necessary force for the stream. One of the advocates of this proceeding mentions at the end of his article that perhaps the method, unless in the hands of the very experienced operator, may do more harm than good, and is perhaps better left alone.

A. OGILVY.

